# Sleep Interruptions Among Older Adults Admitted to the Hospital

**DOI:** 10.1001/jamanetworkopen.2025.1131

**Published:** 2025-03-19

**Authors:** Adrian D. Haimovich, Suzanne M. Bertisch, Venkat Jegadeesan, Jennifer P. Stevens, Mara A. Schonberg, Sarah D. Berry

**Affiliations:** 1 Department of Emergency Medicine, Beth Israel Deaconess Medical Center, Boston, Massachusetts; 2 Department of Medicine, Brigham and Women’s Hospital, Boston, Massachusetts; 3 Beth Israel Lahey Health, Boston, Massachusetts; 4 Department of Medicine, Beth Israel Deaconess Medical Center, Boston, Massachusetts; 5 Center for Healthcare Delivery Science, Beth Israel Deaconess Medical Center, Boston, Massachusetts; 6 Hinda and Arthur Marcus Institute for Aging Research, Hebrew SeniorLife, Boston, Massachusetts

## Abstract

This cohort study examined the length of uninterrupted sleep and types of sleep interruptions in older adults admitted to the hospital through the emergency department.

## Introduction

Sleep deprivation is common among hospitalized older adults and is associated with adverse effects.^1,2^ Hospital sleep barriers include environmental factors, patient conditions (eg, discomfort), and clinical care (eg, vital signs). Using data from electronic health records (EHRs), this study aims to characterize sleep interruptions for clinical care among older adults admitted from the emergency department (ED) within a large health care network.

## Methods

This cohort study included patients aged 65 years or older admitted via the ED to medical-surgical beds at 6 hospitals (1 urban tertiary care, 1 suburban tertiary care, and 4 community) within the Beth Israel Lahey Health Network from June 1 to December 31, 2024. A sleep window from 9 pm to 5 am was defined for each hospitalization night, beginning after an ED clinician requested a hospital bed. Nights with incomplete data (bed request after 9 pm or discharge before 5 am) were excluded. Patient demographics and sleep interruptions—vital signs, medication administrations, imaging, or room changes—were identified using EHRs (eMethods in Supplement 1). The primary outcome was the maximum uninterrupted sleep window during hospitalization nights 1 to 6. Secondary outcomes included (1) percentage of maximum sleep windows at least 7 hours in duration in alignment with current sleep guidelines,^3^ (2) number of sleep interruptions per night, and (3) percentage of interruptions stratified by type of interruption. We stratified sleep duration and interruptions on hospitalization night 1 by whether patients boarded in the ED for 3 hours or more after bed request in accordance with the Age-Friendly Hospital Quality Measure.^4^ Beth Israel Deaconess Institutional Review Board deemed the study exempt with a waiver of informed consent because it was considered secondary research. We followed STROBE reporting guidelines. Two-sided *t* tests were performed, with *P* < .05 considered significant. Data were analyzed with Python 3.10.4 (Python Software Foundation).

## Results

**Figure 1.**
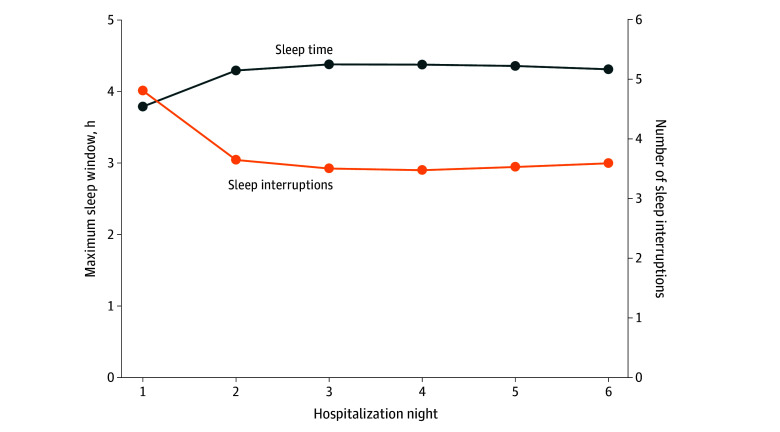
Number of Sleep Interruptions and Maximum Sleep Window by Hospitalization Night

**Figure 2.**
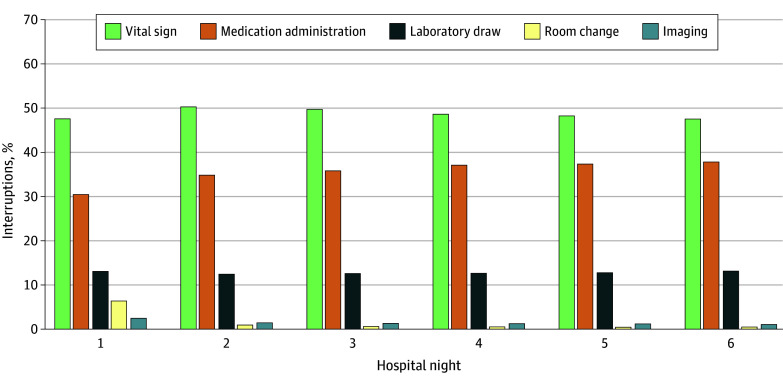
Proportion of Overnight Interruptions by Clinical Task by Hospitalization Night

We included 19 017 patient admissions, accounting for 73 151 patient-nights. Mean (SD) patient age was 78.8 (8.6) years; 10 201 patients [53.6%] were female, with EHR-documented races and ethnicities as follows: 3.1% Asian, 6.6% Black, 4.0% Hispanic or Latino, and 85.8% White. Mean maximum sleep window was 3.8 (95% CI, 3.7-3.8) hours on hospitalization night 1, 4.3 (95% CI, 4.3-4.3) hours on night 2, and 4.3 (95% CI, 4.3-4.4) hours on night 6 (*P* < .001 for night 1 vs nights 2 and 6) (Figure 1). Percentage of maximum sleep windows at least 7-hours long was 2.9% (95% CI, 2.7-3.2) on night 1, 5.4% (95% CI, 5.1-5.7) on night 2, and 5.3% (95% CI, 4.4-5.8) on night 6 (*P* < .001 for night 1 vs nights 2 and 6). Mean number of sleep interruptions was 4.9 (95% CI, 4.8-4.9) on night 1, 3.7 (95% CI, 3.6-3.7) on night 2, and 3.6 (95% CI, 3.5-3.7) on night 6 (*P* < .001 for night 1 vs nights 2 and 6). The most common sleep interruption was checking vital signs, followed by medication administration (Figure 2). On night 1, 65.1% of patients boarded 3 hours or more in the ED and had a mean maximum sleep window of 3.7 (95% CI, 3.7-3.7) hours; those who boarded less than 3 hours had a mean maximum sleep window of 3.9 (95% CI, 3.9-4.0; *P *< .001). Patients boarding in the ED had more interruptions (4.9 [95% CI, 4.9-5.0] vs 4.6 [95% CI, 4.6-4.7]; *P* < .001), with similar proportions of patients having at least 7 hours of uninterrupted sleep (2.8% [95% CI, 2.6%-3.1%] vs 3.4% [95% CI, 2.9%-3.8%]; *P* =.76).

## Discussion

Sleep disruptions were common, particularly on night 1, and were exacerbated among older patients boarding in the ED. Increased interruptions on night 1 may reflect ongoing evaluation. Across nights, vital signs were the primary interruption type. Quality improvement initiatives targeting clinical interruptions with multimodal rest-promoting interventions have not substantially altered sleep windows and patient satisfaction.^5,6^

Study limitations include reliance on timestamps to track interruptions, limited capture of diverse sleep disturbance types, absence of patient acuity measures, and use of single time points for imaging. Further research is needed to evaluate and mitigate hospital sleep disruption and its association with adverse patient outcomes.

## Notes

Supplement 1.
eMethods

Supplement 2.
Data Sharing Statement
