# Supplementary material for: Sleep Interruptions Among Older Adults Admitted to the Hospital
Source: JAMA Netw Open. 2025 Mar 19;8(3):e251131. doi: 10.1001/jamanetworkopen.2025.1131 (PMC11923702; doi:10.1001/jamanetworkopen.2025.1131)
Supplement: Supplement 1. — eMethods [file jamanetwopen-e251131-s001.pdf]

Supplemental Online Content

Haimovich AD, Bertisch SM, Jegadeesan V, Stevens J, Schonberg MA, Berry SD. Sleep interruptions among older adults admitted to the hospital. *JAMA Netw Open.* 2025; 8(3) e251131. doi: 10.1001/jamanetworkopen.2025.1131

eMethods

This supplemental material has been provided by the authors to give readers additional information about their work.

© 2025 Haimovich AD et al. *JAMA Network Open*.

eMethods

Overnight inclusion:

The study defined a sleep window from 9 PM to 5 AM, with an overnight included in the analysis only if the patient was admitted to the hospital for the entire duration. For example, if a patient bed request (i.e., admission) was placed at 2 AM, the first night of hospitalization began at 9 PM that same calendar day. To be included in the study, a patient had to have at least one complete overnight.

Data extraction:

We extracted data from our enterprise data warehouse using custom SQL queries.

Event processing and analysis:

For vital sign timepoints we selected blood pressure recordings, noting that these are more invasive than pulse oximetry and telemetry. For laboratory results, we used the collection instant field as our event timestamp. For imaging, which included electrocardiograms, we used the midpoint of imaging start and ending timestamps as the interruption time. For room changes, we used the timestamp when the patient entered the new room as documented in the EHR. Clustered interruptions within 10 minutes are treated as a single event. All analyses were performed in Python (3.10.4).

© 2025 Haimovich AD et al. *JAMA Network Open*.
