# Supplementary material for: Sleep Interruptions Among Older Adults Admitted to the Hospital
Source: JAMA Netw Open. 2025 Mar 19;8(3):e251131. doi: 10.1001/jamanetworkopen.2025.1131 (PMC11923702; doi:10.1001/jamanetworkopen.2025.1131)
Supplement: Supplement 2. — Data Sharing Statement [file jamanetwopen-e251131-s002.pdf]

Data Sharing Statement

Haimovich. Sleep Interruptions Among Older Adults Admitted to the Hospital. *JAMA Netw Open*. Published March 19, 2025. doi:10.1001/jamanetworkopen.2025.1131

Data
Data available: No

Additional Information
Explanation for why data not available: Not permitted due to PHI.
